# Antibiotic utilization pattern in treatment of acute diarrheal diseases: the case of Hiwot Fana Specialized University Hospital, Harar, Ethiopia

**DOI:** 10.1186/s40545-023-00568-7

**Published:** 2023-05-10

**Authors:** Beyene Dereje, Shegaye Yibabie, Zenebe Keno, Alemayehu Megersa

**Affiliations:** 1 Pharmacology Department, School of Medicine, College of Medicine and Health Science, Dire Dawa University, Dire Dawa, Ethiopia; 2 Medicine Department, School of Medicine, College of Medicine and Health Science, Dire Dawa University, Dire Dawa, Ethiopia; 3 Pharmacy Department, Hiwot Fana Specialized University Hospital, College of Health and Medical Science, Haramaya University, Harar, Ethiopia

**Keywords:** Diarrhea, Antibiotics utilization, Acute diarrheal disease, Cross-sectional study

## Abstract

**Background:**

The vast majority of acute diarrheal diseases are self-limiting and do not require treatment on a regular basis. Empirical antibiotics should only be used to treat dysenteric and invasive bacterial diarrhea. Antibiotic misuse in the treatment of acute diarrhea is widespread in clinical practice worldwide. Hence, the purpose of this study was to examine the pattern of antibiotic use for the acute diarrheal diseases at Hiwot Fana Specialized University Hospital, Harar, Ethiopia.

**Methods:**

A retrospective, institution-based cross-sectional study was conducted to investigate the antibiotic utilization pattern for the treatment of acute diarrheal diseases from September 1 to September 30, 2022. Data were obtained retrospectively from patient cards treated for diarrheal disorders from August 1, 2021 to August 31, 2022, using standardized questionnaires, and the analysis was performed using IBM SPSS Statistics version 27.

**Results:**

Among 332 patients in present study, 271 (81.63%) of them received nine different types of antibiotics, with the most commonly prescribed drugs were Cotrimoxazole (30.26%), Ciprofloxacin (19.19%), and Azithromycin (17.71%). Based on the presence of blood in the stools, 14.76% of the cases were invasive bacterial in nature. Antibiotics were prescribed about 2.55 times more frequently to patients under the age of 12 than to subjects 65 and older (AOR 2.55, 95% CI 1.45–3.87). Patients who received three or more medications were 2.77 times more likely to be prescribed antibiotics (AOR 2.77, 95% CI 1.84–7.56). For every unit increase in the number of drugs prescribed, the odds of prescribing antibiotics increased by 2.44 units (COR 2.44; 95% CI 2.06–4.32).

**Conclusions:**

The current study found that antibiotics were overused in both adults and children with acute diarrheal diseases at Hiwot Fana Specialized University Hospital. The number of antibiotics prescribed was significantly associated with the patient’s age and the number of medications prescribed. To reduce antibiotic overuse, health professionals have to follow the national standard treatment guidelines.

## Background

Antibiotics are medicines that are currently used worldwide to treat bacterial infections in both humans and animals [1, 2]. They work by killing the bacteria or making it difficult for bacteria to proliferate and flourish [3, 4]. A new antibiotic is brought onto the market frequently, leaving doctors little time to thoroughly familiarize themselves with the new medications while also allowing microbes plenty of opportunities to evolve various forms of resistance to secure their survival [5]. Antibiotics can be lifesaving in the treatment of bacterial infections and are the most commonly prescribed drugs among all medications. Their indiscriminate use increases the risk of antibiotic resistance, necessitating more cautious prescribing for the treatment of bacterial infections [6–8].

Antibiotics agent misuse raises therapy costs, adverse drug reactions (ADRs), and patient mortality [9]. Inappropriate antibiotic use is defined as using antibiotics in a way that minimizes the therapeutic effects while increasing toxicity and resistance development. In Ethiopia, there is evidence of antibiotic misuse by healthcare providers, unskilled practitioners, and drug consumers. These, together with the rapid spread of resistant bacteria and insufficient surveillance, will exacerbate the problem [10, 11]. Several studies have found various types of antibiotic misuse in hospital settings in both developing and developed countries, which raises the costs of treating bacterial infections and increases antibiotic resistance [12–16].

Diarrhea is regarded the passing of three or more loose or liquid stools per day. The passing of formed stools on a regular basis is not diarrhea, nor is the passing of loose, “pasty” stools by breastfed babies [17]. There are three distinct clinical kinds of diarrhea: acute bloody diarrhea, commonly known as dysentery; acute watery diarrhea, which lasts several hours or days; and persistent diarrhea, which lasts 14 days or longer [18]. Viruses are the main cause of acute diarrhea both in developed and underdeveloped nations, especially during the winter. No matter the etiology or severity of the process, supportive rehydration therapy is the cornerstone of treatment, and its fast and early adoption is linked to a positive outcome. It should also be combined with proper nutritional support [7, 19].

Since pathogens cannot be identified in more than 90% of diarrhea cases, empirical antibiotic therapy is advised. The clinical value of empiric antibiotic therapy should, however, be evaluated against the risk of side effects and the possibility of removing healthy bacteria [20]. Resistance is frequently linked to increased antibiotic use in hospitals. The rates of resistance shown in multidrug-resistant nosocomial infections are significantly influenced by the usage patterns of antibiotics [21–23]. The expense of treatment has gone up along with the increased morbidity and mortality in many patients due to the rising resistance [10, 22]. The ability of the underprivileged population to access contemporary healthcare will unquestionably be compromised by rising healthcare costs. Furthermore, most hospitals in developing countries had a higher than 30% rate of improper antibiotic use [24].

Antibiotic use is estimated to be inappropriate in 20–50% of cases, according to estimates [25]. This leads to more side effects, higher costs, and a high rate of antibiotic resistance (AMR) in community infections [25]. In severe diarrhea, antibiotics are most commonly misused for viral and self-limiting illnesses. About 70–80% of all diarrheal episodes are caused by viral infections, such as rotavirus [26]. Due to the self-limiting nature of acute diarrhea, complexity and length of time required to identify the pathogen, routine use of antibiotics is not advised in the majority of cases [27]. In a joint statement released in 2004, the World Health Organization (WHO) and the United Nations International Children’s Emergency Fund (UNICEF) suggested treating severe diarrhea in children with the low-osmolality oral rehydration solution (ORS) and zinc tablet [28].

Only in cases of serious bloody diarrhea or dysentery are antibiotics advised. Unfortunately, reports from several parts of the world indicated that improper use of antibiotics in the management of diarrhea is widespread [29]. To support the implementation of antibiotic stewardship programs (ASP) in various healthcare settings, antibiotics utilization pattern indicators could be assessed as useful standards [30–32]. For the purpose of developing a regional intervention program to encourage responsible use of antibiotics, prevent the spread of Antimicrobial Resistance (AMR), and lower the cost of acute diarrhea therapy, it is critical to understand the scope and pattern of antibiotic use for acute diarrhea in the community. As a result, this study was carried out at Hiwot Fana Specialized University Hospital, to analyze the pattern of antibiotic use for the treatment of acute diarrheal diseases.

## Methods

### Study setting and period

This study was carried out at Hiwot Fana Specialized University Hospital, a comprehensive teaching hospital for Haramaya University located in Harar town, 526 km to the east of Ethiopia’s capital, Addis Ababa. It is now the primary teaching and referral hospital in the country’s eastern region. Internal medicine, gynecology, obstetrics, surgery, dentistry, antenatal care, ophthalmology, hospital pharmacy, dermatology, and an antiretroviral therapy clinic are among the services provided by the hospital. From September 1 to September 30, 2022, a cross-sectional study design was used to assess the antibiotics utilization pattern for the management of acute diarrheal diseases in this hospital.

### Study design

An institution-based cross-sectional study was conducted retrospectively, using quantitative approach to assess antibiotic utilization pattern for the treatment of acute diarrheal disease.

### Source population

The source was all diarrheal patient records at Hiwot Fana Specialized University Hospital.

### Study population

The study included patient charts used for the diagnosis and treatment of acute diarrheal disease at Hiwot Fana Specialized University Hospital from August 1, 2021 to August 31, 2022.

### Sample size determination and sampling technique

To obtain the largest possible minimum sample size for this study, it was calculated using the single population proportion formula, assuming a 95% confidence interval, a 5% margin of error, and a prevalence of 50% and calculated with following formula:

$$n = \frac{{\left( {Z_{{1 - {\raise0.7ex\hbox{$\alpha $} \!\mathord{\left/ {\vphantom {\alpha 2}}\right.\kern-0pt} \!\lower0.7ex\hbox{$2$}}}} } \right)^{2} P\left( {1 - P} \right)}}{{d^{2} }} = \frac{{\left( {1.96} \right)^{2} \left( {0.5} \right) \left( {0.5} \right)}}{{\left( {0.5} \right)^{2} }} = 384,$$

where *n* = sample size, *Z*_1−*α*/2_ = standard normal variable at (1 − *α*) % confidence level and *α* (level of significance) was taken to be 5% (95% confidence level is used = 1.96), *P* = prevalence rate estimate for the population (50%), *d* = margin of the tolerated sampling error (0.5)

As a result, the n value was calculated and found to be around 384. The number of medical cards (population size, *N*) of patients who were treated for acute diarrheal disease within study period was 3752. Since the population size was less than 10,000 (*N* = 3752), a reduction formula was utilized using STAT CALC of Epi Info software and the actual sample size was found to be about 332. A systematic sampling technique was used to identify the patient charts. The sampling interval was determined by dividing the total number of patient charts by the sample size, yielding the interval (*k* = 11), and every 11th chart was selected. The first patient chart was chosen by lottery from the first to the eleventh patient chart, based on the time order of the records.

### Data collection tools and procedures

Data abstraction formats were used to collect data retrospectively. The information acquired included the patients’ sociodemographic and clinical features, as well as patterns of antibiotic use over the study period. The patient chart, laboratory data, and medications were all utilized, as well as the prescriber profile. The data collection approach includes essential points that can quantitatively address main drug usage issues during antibiotic use. Every relevant fact was captured in the patient’s medication records.

### Study variables

Explanatory variables included gender, age, duration, prescriber’s profession, and laboratory tests of stool characteristics, while the dependent variable was antibiotic utilization pattern.

### Data processing and analysis

IBM SPSS Statistics version 27 was used to process and analyze the collected data. To provide the frequency and percentage distributions of the variables included in the study, descriptive statistics were used, followed by cross-tabulation. The outcome was presented in the form of narratives, tables, and figures.

### Data quality control

A pretest was performed at the Jinela Health Center to determine whether the data collection format was valid and reliable, and the completeness of the data collection format was checked prior to the actual data collection. Data cleaning was also performed accordingly.

## Results

### Sociodemographic characteristics of the patients

There were 3752 patient records documented as diagnosed with acute diarrheal diseases within study period (August 1, 2022 to August 31, 2022). A total of 332 patient records were included in the study. Among 332 patients, 183 (55.12%) were males and 149 (44.88%) were females. Children under 5 years of age were 48.80% and patients > 65 years were 6.63% (Table 1).

**Table 1:** Sociodemographic characteristics of the acute diarrhea patients at Hiwot Fana Specialized University Hospital; August 1, 2021 to August 31, 2022

Study variables	Frequency	Percentage
Sex of patients		
Male	183	55.12
Female	149	44.88
Age of patients		
< 5 years	162	48.80
5–12 years	75	22.59
13–40 years	38	11.45
41–65 years	35	10.54
> 65 years	22	6.63

### Clinical characteristics

**Fig. 1:**
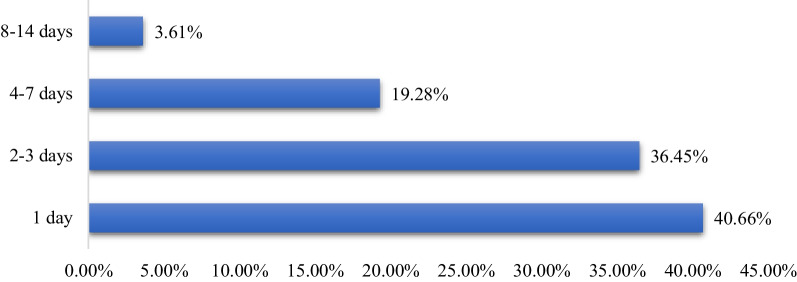
Duration of diarrhea from onset to treatment for patients diagnosed with acute diarrhea at Hiwot Fana Specialized University Hospital; August 1, 2021 to August 31, 2022

A review for the history of the cases shows that, 121 (36.45%) patients had experienced the illness for 2–3 days (Fig. 1). Most patients reported diarrhea-related illnesses, such as fever 142 (42.77%), vomiting 194 (58.43%), cough 23 (6.93%), chills 7 (2.11%), headache 29 (8.74%), abdominal cramps 109 (32.83%) and loss of appetite 26 (7.83%). From all patients, 84 (25.31%) of them had mild to moderate dehydration, while six patients (1.81%) had severe dehydration, which required intravenous fluid therapy.

### Stool characteristics

**Fig. 2:**
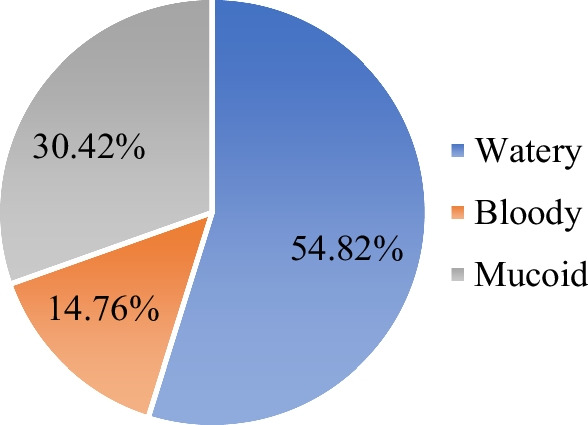
Stool characteristics of patients diagnosed with the acute diarrhea at Hiwot Fana Specialized University Hospital; August 1, 2021 to August 31, 2022

From 332 patients, 237 (71.38%) patients had a stool examination ordered and 119 (50.21%) stool specimens were recorded positive as 81 (34.18%) were with unspecified bacteria and 57 (24.05%) contain amoeba, giardia, and ascariasis. Majority, 85.24% of stools were non-bloody and 14.76% have blood in stools (Fig. 2). The percentage of patients with bloody diarrhea that has received antibiotics was (100%), watery (86.26%) and mucoid (64.36%) (Table 2).

**Table 2:** Antibiotics prescription by age groups and stool characteristics for acute diarrhea patients at Hiwot Fana Specialized University Hospital; August 1, 2021 to August 31, 2022

Antibiotics prescribed	Age groups	Watery (*N* = 182)		Bloody (*N* = 49)		Mucoid (*N* = 101)	
		Frequency	%	Frequency	%	Frequency	%
Yes (*N* = 553)	< 5	83	45.60	20	40.82	33	32.67
	5–12	31	17.03	10	20.41	16	15.84
	13–40	17	9.34	5	10.20	9	8.91
	41–65	14	7.69	8	16.33	4	3.96
	> 65	12	6.59	6	12.24	3	2.97
	Sub-total	157	86.26	49	100.00	65	64.36
No (*N* = 73)	< 5	14	7.69	0	0.00	12	11.88
	5–12	5	2.75	0	0.00	13	12.87
	13–40	2	1.10	0	0.00	5	4.95
	41–65	4	2.20	0	0.00	5	4.95
	> 65	0	0.00	0	0.00	1	0.99
	Sub-total	25	13.74	0	0.00	36	35.64
Total		182	54.82	49	14.76	101	30.42

*N* number of patients/records

### Treatment patterns of acute diarrheal diseases

**Fig. 3:**
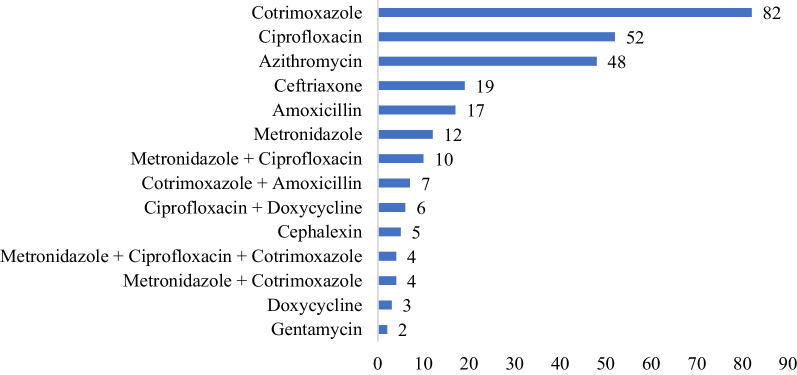
Antibiotics prescribed for the acute diarrhea patients at Hiwot Fana Specialized University Hospital; August 1, 2021 to August 31, 2022

The patient’s record shows that, the number of antibiotics prescribed for single patient ranged from 1 to 3 drugs. About 81.63% of cases received at least one antibiotic drug, while 18.37% of them received no antibiotics. Specifically, 72.28% of patients received one, 8.13% received 2, and 1.21% received 3 antibiotics during the episode of diarrhea. There are nine types of antibiotics that prescribed for acute diarrhea treatment. Cotrimoxazole (30.26%), Ciprofloxacin (19.19%), Azithromycin (17.71%), Ceftriaxone (7.01%) and Amoxicillin (6.27%) were the most frequently prescribed antibiotics. In the same manner, of 162 under five children, 136 (83.95%) were prescribed with at least one antibiotic (Table 5). Of 332 patients, 151 (49.83) patients were prescribed with ORS, while 6 patients prescribed with IV fluid for treatment of dehydration. Other medications prescribed were; Paracetamol 139 (41.87%), Albendazole 23 (6.93%), Mebendazole 17 (5.12%), Ibuprofen 16 (4.83%), Diclofenac 14 (4.22%), Multivitamin 11 (3.31%), Tramadol 10 (3.01%), Metoclopramide 9 (2.71%), Omeprazole 7 (2.11%), Tinidazole 7 (2.111%), and Hyoscine 5 (1.51%) (Fig. 3).

### Adherence to standard treatment guidelines

The adherence to Standard Treatment Guideline (STG) was routinely assessed to show the appropriateness of antibiotics prescribing pattern. The result shows that 116 (34.94%) cases were treated in line with STG, while 216 (65.06%) cases were not treated according to National Standard Treatment Guideline recommendations (Table 3).

**Table 3:** Antibiotic utilization patterns based on STG for acute diarrheal diseases in Hiwot Fana Specialized University Hospital; August 1, 2021 to August 31, 2022

Antibiotic usage	Prescribed in line with STG		Not prescribed in line with STG	
Given antibiotic for bloody diarrhea	49	14.76%	0	0.00%
Given IV fluid for severe diarrhea	6	1.81%	0	0.00%
Given antibiotic for non-bloody diarrhea	0	0.00%	216	65.06%
Not given antibiotic for non-bloody diarrhea	61	18.37%	0	0.00%
Total	116	34.94%	216	65.06%

*IV* intravenous, *STG* Standard Treatment Guideline

### Prescriber profile on acute diarrheal diseases

Most of the acute diarrheal patients were treated by Medical Interns, 149 (44.88%), General Practitioners, 61 (18.37%) and Nurses, 61 (18.37%). About 44 (13.25%) of patient records had no name of prescribers. The better proportion of antibiotic prescription in line with STG was among Senior Physicians (64.71%) and General Practitioners (55.74%), while Nurses (75.41%) and Medical Interns (65.77%) prescriptions were not in line with National Standard Treatment Guideline recommendations (Table 4).

**Table 4:** Antibiotic usage for the acute diarrheal diseases of health professionals in Hiwot Fana Specialized University Hospital; August 1, 2021 to August 31, 2022

Prescriber profession	Frequency	%	Antibiotic utilization pattern			
			In line with STG		Not in line with STG	
			Frequency	%	Frequency	%
Specialist (MD)	17	5.12	11	64.71	6	35.29
General practitioners (GP)	61	18.37	34	55.74	27	44.26
Medical intern (MD)	149	44.88	51	34.23	98	65.77
Nurse	61	18.37	15	24.59	46	75.41
Unknown	44	13.25	5	11.36	39	88.64
Total	332		116		216	

*MD* medical doctor, *STG* Standard Treatment Guideline

### Antibiotic prescribing predictors

At the bivariate level, the predictors of antibiotic prescribing, age (*P* = 0.013) and number of medicines prescribed (*P* < 0.006), were significantly associated with antibiotic prescribing. Antibiotic drugs were 2.46 times more likely to be given to patients under the age of 12 than to patients 65 and older (AOR 2.46, CI 1.23–4.36). When compared to those who received one or two antibiotics per prescription, those who received three or more drugs per prescription were more likely to receive an antibiotic. Hence, patient taking four drugs have more than three times probability of antibiotic prescribed for them (AOR 3.25, CI 1.51–33.52) (Table 5).

**Table 5:** Bivariate analysis of predictors of the prescribed antibiotics for acute diarrheal disease in Hiwot Fana Specialized University Hospital; August 1, 2021 to August 31, 2022

Variables	Antibiotic prescribed				Bivariate analysis	
	No	Percent	Yes	Percent	COR (95% CI)	*P* value
Age of patients						0.013
< 5 years	26	16.05	136	83.95	2.17 (1.61–3.92)	0.001
5–12 years	18	24.00	57	76.00	2.46 (1.23–4.36)	0.003
13–40 years	7	18.42	31	81.58	1.04 (0.54–1.20)	0.142
41–65 years	9	25.71	26	74.29	1.01 (0.33–1.14)	0.113
> 65 years	1	4.55	21	95.45	Ref.	
Sex of patients						
Male	14	7.65	169	92.35	1.03 (0.81–1.25)	0.136
Female	47	31.54	102	68.46	Ref.	
Number of medication prescribed						0.006
1 drug	38	84.44	7	15.56	Ref.	
2 drugs	8	8.51	86	91.49	0.79 (0.26–1.27)	0.132
3 drugs	13	9.42	125	90.58	2.48 (1.39–22.31)	0.002
4 drugs	2	4.26	45	95.74	3.25 (1.51–33.52)	0.001
5 drugs	0	0.00	8	100.00	7.11 (1.79–36.48)	0.017

*COR* crude odd ratio, *CI* confidence interval

*P < 0.05 was considered significant

The full analysis model fitness test was performed to confirm the suitability and found analysis model containing all predictors was statistically significant, *χ*^2^ (5, *N* = 332) = 76.95, *P* < 0.001, indicated that the model was able to distinguish between the respondents who had been prescribed antibiotics and those who had not. Hosmer and Lemeshow test also supported the model fitness (*χ*^2^ = 6.382, *df* = 6, *P* = 0.613). The model as a whole also explained between 58.4% (Cox and Snell *R* square) and 78.1% (Nagelkerke *R* square) of the variance in antibiotic prescription and correctly classified 64.33% of those who had one. According to the model’s sensitivity, it correctly identified 59.6% of the group with antibiotic prescribed. Furthermore, the specificity was 67.4%. Age (*P* = 0.011) and number of medicines prescribed (*P* < 0.002) significantly associated with antibiotic prescribing after adjusting for potential confounders using multivariate logistic regression (Table 6). There is a significant increase in antibiotic prescribing with an increase in the number of medicines prescribed (*P* < 0.002). The odds of prescribing antibiotics increased by 2.44 units for every unit increase in the number of medicines prescribed (COR 2.44; 95% CI 2.06–4.32).

**Table 6:** Multivariate analysis of predictors of the prescribed antibiotics for acute diarrheal disease in Hiwot Fana Specialized University Hospital; August 1, 2021 to August 31, 2022

Study variable	Multivariate analysis	
	COR (95% CI)	*P* value
Age of patients		0.011
< 5 years	2.31 (1.73–3.12)	0.001
5–12 years	2.55 (1.45–3.87)	0.014
13–40 years	1.04 (0.54–1.20)	0.142
41–65 years	1.01 (0.33–1.14)	0.113
> 65 years	Ref.	
Sex of patients		
Male	1.06 (0.84–1.27)	0.141
Female	Ref.	
No of medication prescribed		0.002
1–2 drugs	Ref.	
3–4 drugs	2.77 (1.84–7.56)	< 0.001
5 or more drugs	6.51 (1.89–47.22)	0.012

*COR* crude odd ratio, *CI* confidence interval

*P < 0.05 was considered significant

## Discussion

### General prescribing pattern

This institution-based cross sectional study has investigated the pattern of antibiotic use for acute diarrheal diseases in Hiwot Fana Specialized University Hospital, Harar, Ethiopia. In the present study, 81.63% of patients have received at least one antibiotic drug. This result is slightly lower than study done at Bishoftu General Hospital, Ethiopia which was 86.8% [33] and far higher than the findings of the studies carried out in different parts of the world such as India with 71% [34], China 60.8% [35], and Thailand 45.1% [36] that had received an antibiotic drug for acute diarrheal disease. There could be a number of causes for the high prescription rate for antibiotics. The high level of routine empirical treatments observed in resource-poor nations is primarily a result of the increased occurrence of infectious diseases in developing countries, which increases the number of antibiotics prescribed [37]. The other factor can be patient pressure on doctors [38].

Antibiotic self-medication was reported to be common and about 44–45.1% in Ethiopia and Eritrea, according to several studies and a comprehensive review [39–41]. This finding may indicate that patients are more likely to directly or indirectly request antibiotic prescriptions from doctors as they are heavily involved in self-medicating with antibiotics [42]. The trend of King Chulalongkorn Memorial Hospital, Thailand with better prescribing pattern may be due to advanced practice and knowledge toward antibiotics rational use, enhanced education and control of over the counter drugs and better trend of following the standard treatment guideline recommendations [36].

For the 332 patients treated for acute diarrhea that were included in the current study, a total of 737 medications were prescribed, resulting in an average of 2.72 drugs per prescription, which is similar to study done in south India with 2.7 [43], but much higher than the WHO standard (1.6–1.8) [44], as well as some results from the comparable investigations carried out across Ethiopia, which revealed an average of 1.64–1.90 medications per encounter [45–52]. However, when compared to several other study results from Ethiopia, Sudan, India, and Saudi Arabia, which were found an average value of 2.02–4.2 medicines per encounter, this number is the comparable one [30, 31, 53–60]. This shows that, prescribers should restrict medicine prescriptions to only patients that are absolutely essential, because polypharmacy can expose patients to unfavorable drug effects and raise patient costs.

The percentage of encounters in this study when at least one antibiotic was prescribed was 41.52%, which is much higher than the WHO standard value of 20–26.2%. This result is comparable with study done in Bahawalpur, Pakistan which was 48.6% [61]. Similar studies carried out in various nations indicated that a percentage of encounters with antibiotics were between 9.1 and 38.4%, which is less than the result reported by the current study [31, 43, 47, 48, 56]. On the other hand, the result is lower than those of other comparable studies with a range of 52.3–75.1% [51, 52, 54, 55, 59, 61, 62].

In present study, 83.95% of children under 5 years with acute diarrhea have received at least one antibiotic drug which is lower than study done in Bishoftu General Hospital, Ethiopia with 92.6% [33]. However, higher than the findings of the studies conducted in Central Region Province of Thailand, Delhi, India, and Puducherry, India, where the percentage of patients prescribed on antibiotics were 72.6%, 64%, and 22%, respectively [63–65]. The percentage of acute diarrheal patients treated not in line with STG was 65.06%, which is slightly better than other study done in Bishoftu, Ethiopia with 72.3% [33]. However, the result is higher in percentage than the finding of the study carried out in China at 51.3% [35] and Thailand at 48.9% [36]. However, other study conducted in South Thailand, indicated that 73.8% of antibiotics prescribed were in line with STG for diarrheal disease treatment [66]. The most commonly prescribed drugs for acute diarrheal diseases were Cotrimoxazole (30.6%), Ciprofloxacin (19.19%) and Azithromycin (17.71%) in the present study. The finding is different from other study conducted in Thailand [63] and Ethiopia [33] as both studies indicated greater than 50% prescription was only Cotrimoxazole.

### Antibiotic prescribing predictors

This study discovered a significant correlation between patient age and number of medications for antibiotics prescribed. Antibiotic prescriptions were found to be associated with being under the age of 12 as they got the highest proportion of antibiotics when compared to the other patient categories which is similar to research from Eritrea [40], Bangladesh [67], Yemen [68], and Cameroon [69]. According to the results of the current study, prescribing three or more medications per prescription was highly associated with prescribing antibiotics.

Antibiotics were about 2.55 times more likely to be prescribed to patients under the age of 12 than to subjects of 65 years and older (AOR 2.55, 95% CI 1.45–3.87). When compared to subjects who received one or two drugs per prescription, those who received more than two drugs were 2.77 times more likely to receive an antibiotic (AOR 2.77, 95% CI 1.84–7.56). The odds of prescribing antibiotics were increased by 2.44 units for every one unit increase in the number of medicines prescribed (COR 2.44; 95% CI 2.06–4.32). It is consistent with study done in Asmara, which found that probabilities increased by 2.02 for every one-unit increase (*P* < 0.001; OR 2.02; 95% CI 1.62–2.52) [40] and Zambia, where it was shown that odds rise by 2.7 for every one-unit increase (*P* < 0.001; OR 2.68, 95% CI 2.20–3.25) [70].

The current study limitation is that, it was conducted in only one hospital and so cannot be generalized to other facilities. However, because Hiwot Fana Specialized University Hospital is the only tertiary hospital in the research area, the current study can provide a picture of how antibiotics are used in East Ethiopia. This study discovered a significant incidence of incorrect antibiotic use, which may fuel rising antimicrobial resistance and associated costs on a national and worldwide scale. In general, the study determined the prevalence of antibiotic use, identified the types of antibiotics used in the treatment of acute diarrheal illness, and rated prescribers’ adherence to standard treatment guidelines.

## Conclusion

The present study revealed that there was high overuse of antibiotics for both adults and children with acute diarrheal disease in Hiwot Fana Specialized University Hospital. The most common antibiotics prescribed were Cotrimoxazole, Ciprofloxacin and Azithromycin. The proportion of prescriptions containing an antibiotic was 41.52%, which is much higher than WHO-recommended standard (20–26.2%). The average number of prescriptions per encounter fell just short of WHO recommendations, and adherence to the Standard Treatment Guideline (STG) was also inadequate. Antibiotic prescribing revealed a strong correlation with patient age and the number of medications per prescription. Thus, to reduce antibiotics overuse, health professionals have to follow the national standard treatment guidelines.

## Data Availability

On reasonable request, the data used and analyzed during this study can be obtained from the corresponding author.

## Abbreviations

ADR: Adverse drug reaction
AMR: Antimicrobial resistance
ASP: Antimicrobial Stewardship Program
EML: Essential Medicine List
ORS: Oral rehydration salt
STG: Standard treatment guideline
UNICEF: United Nations International Children’s Emergency Fund
WHO: World Health Organization
